# Retraction: Triple Reassortant Swine Influenza A (H3N2) Virus in Waterfowl

**DOI:** 10.3201/eid1901.C11901

**Published:** 2013-01

**Authors:** Sagar M. Goyal

**Affiliations:** Author affiliation: College of Veterinary Medicine– University of Minnesota, St. Paul, Minnesota, USA

**Keywords:** Influenza A virus, triple reassortant, H3N2, waterfowl, interspecies transmission, viruses

**To the Editor:** We would like to retract the letter entitled “Triple Reassortant Swine Influenza A (H3N2) Virus in Waterfowl,” which was published the April 2010 issue of Emerging Infectious Diseases (*1*). The nucleoprotein gene sequences from the viruses reported in that letter are very closely related to those from the earliest detected triple reassortant swine influenza viruses [CY095676 A/sw/Texas/4199–2/1998(H3N2)]. Although these viruses could have acquired a swine-origin segment, the branch lengths are quite short for 9 years of evolution. Therefore, we have withdrawn these 4 isolates from GenBank and subsequently retract this letter.
